# Current evidence regarding alternative techniques for enterocystoplasty using regenerative medicine methods: a systematic review

**DOI:** 10.1186/s40001-024-01757-z

**Published:** 2024-03-12

**Authors:** Razman Arabzadeh Bahri, Maral Peisepar, Saba Maleki, Fatemeh Esmaeilpur Abianeh, Fatemeh A. Basti, Ali Kolahdooz

**Affiliations:** 1 Urology Research Center, Tehran University of Medical Sciences, Tehran, Iran; 2 Universal Scientific Education and Research Network (USERN), Tehran, Iran; 3 School of Medicine, Tehran University of Medical Sciences, Tehran, Iran; 4 School of Medicine, Guilan University of Medical Sciences, Rasht, Iran; 5 Tehran Medical Branch, Islamic Azad University, Tehran, Iran; 6 Student Research Committee, School of Medicine, Alborz University of Medical Sciences, Karaj, Iran

**Keywords:** Regenerative medicine, Urinary bladder, Urologic surgical procedures

## Abstract

Enterocystoplasty is the most commonly used treatment for bladder reconstruction. However, it has some major complications. In this study, we systematically reviewed the alternative techniques for enterocystoplasty using different scaffolds. A comprehensive search was conducted in PubMed, Embase, and Cochrane Library, and a total of 10 studies were included in this study. Five different scaffolds were evaluated, including small intestinal submucosa (SIS), biodegradable scaffolds seeded with autologous bladder muscle and urothelial cells, dura mater, human cadaveric bladder acellular matrix graft, and bovine pericardium. The overall results revealed that bladder reconstruction using regenerative medicine is an excellent alternative method to enterocystoplasty regarding the improvement of bladder capacity, bladder compliance, and maximum detrusor pressure; however, more large-scale studies are required.

**Supplementary Information:**

The online version contains supplementary material available at 10.1186/s40001-024-01757-z.

## Introduction

Conditions like congenital abnormalities, bladder dysfunction, cancers, and trauma can be destructive to the anatomy and physiology of the bladder [1]. Neurogenic bladder dysfunction, spina bifida, detrusor overactivity, and chronic inflammatory disease of the bladder, such as tuberculosis, schistosomiasis, interstitial cystitis, and bladder pain syndrome, are other conditions affecting bladder function [2]. In these conditions, reduced bladder capacity, incontinence, and renal damage can occur due to bladder exstrophy and neurotrophic bladder [3]. A healthy bladder is essential for a high quality of life. Although in neurogenic bladders, congenital disorders, or inflammatory conditions, when medical treatments are not effective, surgical treatments are usually offered [4]. Enterocystoplasty for neo-bladder creation is the gold-standard treatment for bladder cancer [5, 6]. Enteroplasty is used as the most common urethroplasty procedure in urethral abnormalities, bladder reconstruction, and urinary bladder disorders [7]. Some adverse effects in cystoplasty are reported, including ureteral stricture, dehiscence of the repaired area, and fistulas [3, 8]. Intestinal obstruction, metabolic disturbance, urine leakage, stone formation, and chronic infections are the adverse side effects reported following the enteroplasty surgery for bladder reconstruction [9].

Nowadays, tissue engineering may be a proper solution to the present limitations regarding bladder reconstruction techniques. In tissue engineering, a tissue or an organ is grown through cell implantation or the growth of host cells [10]. Mechanical features similar to the native tissue, easy manipulation, biocompatibility, and negligible or absent immunological reactions are the most important criteria for choosing the ideal scaffold and cell type in seeded grafts [11–15]. Some of the appropriate natural scaffolds in tissue engineering of bladder reconstruction are natural polymers, small intestinal submucosa (SIS), bladder acellular matrix (BAM), amniotic membrane, and dermis [16–20]. Moreover, synthetic scaffolds and hybrid scaffolds produced from the combination of synthetic and natural scaffolds are used in bladder reconstruction during tissue engineering [21, 22]. For instance, a hybrid scaffold that combines polycarbonate urethanes and small intestinal submucosa (SIS) was recently proposed to offer the mechanical strength of the synthetic polymer and the biological advantages of SIS. Utilizing the benefits of each component, the hybrid approach seeks to maximize the properties of the scaffold [23]. Today, determining the best type of stem cells for regeneration and choosing the best materials and technique for inserting these cells are the most controversial topic [24, 25]. Although bladder reconstruction through tissue engineering is a promising procedure, side effects of this procedure, such as strictures, fistulas, early tissue fibrosis, bladder contracture, and inadequate vascularization, are the most challenging topics in the use of this procedure clinically [26–28]. In this study, we aimed to systematically review the studies that performed bladder reconstruction using regenerative medicine methods as alternative techniques for enterocystoplasty.

## Methods

This systematic literature review was performed based on the guidelines of the Cochrane book and Preferred Reporting Items for Systematic Reviews and Meta-Analyses (PRISMA) [29, 30].

### Search strategy

A thorough search was conducted in PubMed, Embase, and Cochrane Library bibliometric databases from the inception of the databases to February 20th, 2023. The keywords that were used were divided into two groups, including bladder reconstruction and regenerative medicine groups. The keywords for the bladder reconstruction group were any possible keywords, such as bladder augmentation, neobladder, or cystoplasty. The keywords for the regenerative medicine group included tissue engineering, cell engineering, cell therapy, or 3D bioprinting. The keywords were searched using “OR” between the keywords of each group and using “AND” the between two groups. The detailed search strategy strings are provided in Additional file 1: Table S1. A second search was conducted one week before the submission in order to identify and include any newly published article regarding the goal of this study on October 10, 2023.

### Eligibility criteria

Any human studies that proposed a method in the field of regenerative medicine for bladder reconstruction except enterocystoplasty were included in this study. Review studies, meta-analyses, and animal studies were excluded. No language restrictions were imposed.

### Data extraction

The titles and the abstracts of identified articles were screened by two independent reviewers based on inclusion criteria. After excluding the irrelevant articles, the full texts of the included articles were assessed by two reviewers. Any disagreements were resolved by a third researcher. An Excel-based sheet was used for data extraction, which was conducted by two independent reviewers. The data sheet included the items, including the name of the first authors, year of publication, country of origin, age of the study participants, sample sizes, gender of the patients, scaffold type, the objective of the studies, the types of the underlying bladder disorder, main findings, follow-up time, follow-up results, and complications.

## Results

### Overview of the studies

**Fig. 1:**
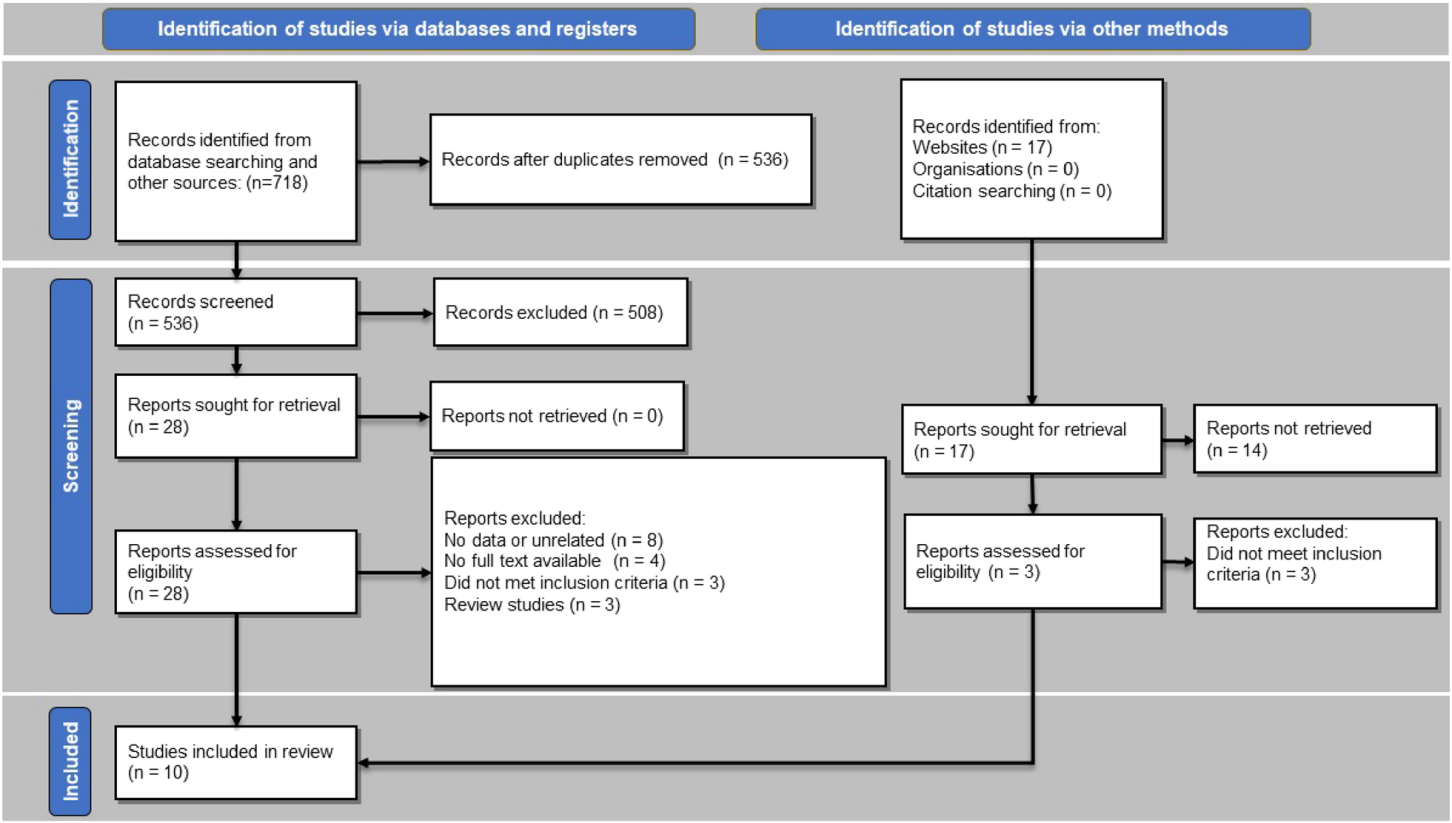
PRISMA flowchart of the literature search and selection of the articles

The comprehensive database search for conducting the present systematic literature review yielded a total of 718 articles. The searched databases included Embase, PubMed, and the Cochrane Library international bibliometric databases. Five hundred and thirty six articles were enrolled for screening after removing duplicate papers. The initial screening of the articles was based on examining the titles and abstracts. A total of 28 articles were selected for screening of their full texts based on our inclusion criteria. Ten studies were included in our study, and their data were extracted (Fig. 1). Eighteen studies were excluded from our second screening phase, which was assessing the full texts of the articles due to improper study design, such as review articles and the unavailability of the full texts. The characteristics of the included articles are presented in Table 1. The 10 included studies were conducted in seven different countries, including the United States with the most articles (*n* = 3) [31–33], China (*n* = 2) [34, 35], Italy (*n* = 1) [36], Brazil (*n* = 1) [37], Turkey (*n* = 1) [38], Germany (*n* = 1) [39], and Korea (*n* = 1) [40]. The studies were published between 1995 and 2019. A total of 69 patients were evaluated in the included studies.

**Table 1:** Baseline characteristics of the included studies

First author	Year of publication	Country	Age: mean (SD) [range]	Sample size	Female (%)	Scaffold type	Seeded/unseeded	Objective	Type of bladder disorder	Main findings	Follow-up time [mean (range)]	Follow-up results	Complications
Zhang	2020	China	29.6	15	7 (44.6)	SIS	Unseeded	Evaluating the long-term effect of SIS for bladder augmentation	Neurogenic bladder	Significant increases in bladder capacity. Significant decrease in maximum detrusor pressure. Immediate failure in two patients. Decreased bladder capacity in four patients	6.3 (4.5–8.3) years	Histology: complete conversion of SIS. The bladder wall contained vessels, and thick connective tissue	Vesicoureteral reflux: *n* = 5, bladder stones: *n* = 1, bladder perforation: *n* = 1
Atala	2006	USA	4–19	7	N/A	Autologous cell-seeded biodegradable scaffold	Seeded	To Engineer bladder tissues by autologous cells	High-pressure or poorly compliant bladders due to myelomeningocele	Decreased bladder leak point pressure at capacity Increased volume and compliance	46 (22–61) months	Adequate structural architecture and phenotype	None
Caione	2012	Italy	10.4	5	2 (40)	SIS	Unseeded	To improve bladder compliance and capacity	Exstrophic bladder	Increased bladder capacity and compliance. Decreased muscle to collagen ratio	3 years	No bladder diverticula, renal damage, or stones	None
Arikan	1995	Turkey	[9–51]	10	4 (40)	Dura mater	Unseeded	To use dura mater for bladder augmentation	Neurogenic bladder and bladder dysfunction	Immediate urinary continence in all patients. Satisfactory continence and urodynamic improvement	N/A	N/A	None
Zhang	2014	China	25.4	8	2 (25)	SIS	Unseeded	To use SIS for bladder augmentation	Poor bladder capacity and compliance	Improvements in maximum bladder capacity and bladder compliance. Decreased maximum detrusor pressure	11–36 months	No metabolic consequence	None
Shenot	2011	USA	28.7	6	2 (33)	Autologous cell-seeded biodegradable scaffold	Seeded	Treatment of neurogenic bladder	Neurogenic bladder	Feasibility of regenerative medicine in bladder augmentation	24 months	Decreased maximum detrusor pressure	Postoperative bladder leaks
Schaefer	2013	Germany	6.5–15.4 (9.8)	6	N/A	SIS	Unseeded	To use SIS for bladder augmentation	Microbladder	Increased bladder capacity	4.6–32.5 months (24.4)	Histology: complete conversion of SIS. The bladder wall contained vessels, thick connective tissue, and smooth muscles	Bladder stones: *n* = 2, bladder rupture: *n* = 1
Ribeiro-Filho	2005	Brazil	52	1	1 (100%)	Human cadaveric bladder acellular matrix graft	Unseeded	To augment a contracted bladder	Short voiding intervals, nocturia, and bilateral ureteral reflux	Improved urination after catheter removal with no further self-catheterization	40 months	Increased voiding intervals and bladder capacity, reduced nocturia,, and normal urinary flow	None
Moon	2010	Korea	67	1	1 (100%)	Bovine pericardium	Unseeded	To perform bladder reconstruction	Enterovesical fistula	Intact scaffold after six months	2.5 years	Intact scaffold	None
Joseph	2014	USA	8.2	10	6 (60%)	Autologous cell-seeded biodegradable scaffold	Seeded	Using a technique as an alternative to traditional enterocystoplasty	Neurogenic bladder	Improved compliance, no improvement in bladder capacity	12 and 36 months	Alteration in compliance and capacity of the bladder	Low cell growth, urinary tract infection, bowel obstruction, and bladder rupture

### Different methods for bladder reconstruction

Five different scaffolds were used in the included studies, including small intestinal submucosa (SIS), biodegradable scaffolds seeded with autologous bladder muscle and urothelial cells, dura mater, human cadaveric bladder acellular matrix graft, and bovine pericardium.

### Small intestinal submucosa (*n* = 4)

Four articles of our included studies evaluated the efficacy of SIS for bladder reconstruction. In a study by Zhang and Liao [34], 15 patients with poor bladder compliance and low capacity secondary to neurogenic disorder were evaluated and planned for bladder augmentation using SIS scaffold. Long-term follow-up results revealed that there was a significantly increased bladder capacity and a significant decrease in maximum detrusor pressure compared with the baseline period. Nine patients (60%) had expected long-term benefits, and four patients (26.6%) showed a slow decrease in bladder capacity. In another study which was conducted by Caione et al. [36], five patients underwent bladder augmentation using SIS due to poor bladder compliance and capacity after complete exstrophy repair. There was a significant increase in bladder compliance and capacity six months after the procedure, compared with the pre-surgery period. On the other hand, dry intervals were not significantly changed. Also, functional results at 18 months’ timeline did not vary significantly from 6-month timeline. At long-term follow-up, a progressive increase in bladder capacity with no urological complication was reported. In another study by Zhang and Liao [35], eight candidates with poor bladder compliance and capacity were enrolled for SIS cystoplasty. Among those, six patients had myelomeningoceles, and two patients had spinal cord injuries. There were significantly increased maximum bladder capacity at three-month and twelve-month follow-ups, compared to the pre-operative period. Also, a significant increase in bladder compliance and a significant decrease in maximum detrusor pressure were noted. Schaefer et al. [39] conducted bladder augmentation in six patients using the SIS scaffold. The patients had suffered from cloacal exstrophy, vesicoureteral reflux, spina bifida, and bladder exstrophy. An increase in the bladder volume of four patients was achieved. However, a conversion of SIS into irregular bladder wall and urothelial lining, and also a thick connective tissue, was observed in four out of six patients.

### Biodegradable scaffolds seeded with autologous bladder muscle and urothelial cells (n = 3)

Three of our included studies had utilized this scaffold for bladder augmentation. In the study by Atala and colleagues [31], the trial was on seven patients with myelomeningocele. The patients had poorly compliant and high-pressure bladders. Three different implant types were used for the patients, including collagen implant (C), collagen plus omental wrap implant (C + O), and composite collagen plus omental wrap implant (PC + O). The bladder compliance increased by 15%, 67%, and 179% postoperatively in the C, C + O, and PC + O implants, respectively. Moreover, the bladder capacity showed a 30% decrease in C plants after the surgery, but 22% and 57% increase in C + O and PC + O implants were reported, respectively. In a phase II study by Joseph et al. [33] in adolescents and children with spina bifida, 10 patients were enrolled for augmentation cystoplasty using an autologous cell-seeded biodegradable scaffold. Due to their methods, after an open bladder biopsy, smooth muscle and urothelial cells were cultured outside of the body and seeded onto a biodegradable scaffold to create a regenerative augment that served as the basis for the regeneration of bladder tissue. The only concurrent surgical procedure that was allowed was a bladder neck sling. After surgery, bladders were cycled to stimulate regrowth. An insignificant improvement in bladder compliance was reported in 4 and 5 patients at 12 months and 36 months postoperative follow-up, respectively. Also, there was no statistically significant improvement in bladder capacity. In another phase II study, Shenot and colleagues [32] performed the implantation of neo-bladder augmentation, which is an autologous cell-seeded biodegradable scaffold in six patients with neurogenic bladder, secondary to spinal cord injury. After an open bladder biopsy, urothelial and smooth muscle cells were grown ex vivo and then seeded onto a biodegradable scaffold to form the NBA. The implanted NBA served as a template for bladder tissue regeneration. A decrease in maximum detrusor pressure was noted. Also, they reported that there were two asymptomatic urine leakages, which were resolved spontaneously, one construct leak secondary to infection, which was resolved by administration of antibiotics, and one bladder perforation during the 9-month follow-up, which resolved with conservative therapy.

### Dura mater (*n* = 1)

Arikan et al. [38] conducted a study on ten patients with neurogenic bladder dysfunction who were unresponsive to conservative management. They used dehydrated human skull dura mater as a scaffold for bladder regeneration. All of the patients achieved urinary continence to different degrees. Seven patients were completely continent postoperatively with clean intermittent catheterization, although three patients required continuation of oral therapy with oxybutynin at a lower dosage compared to the preoperative period.

### Bovine pericardium (*n* = 1)

In a case report by Moon et al. [40], a patient with an enterovesical fistula was planned for bladder reconstruction using a bovine pericardium as a scaffold. The enterocystoplasty was contraindicated in this patient because of poor intestinal state due to prior radiation therapy. Contracted bladder and vesicoureteral reflux remained in the patient but the dye leakage in the cystography was not observed by postoperative week 8. A cystoscopy was performed 2.5 years after the surgery, and the intact bovine pericardium was observed at the dome of the bladder.

### Human cadaveric bladder acellular matrix graft (*n* = 1)

A patient with an overactive contracted bladder underwent a bladder acellular matrix graft augmentation cystoplasty in the study by Ribeiro-Filho et al. [37]. The cadaveric bladder was enzymatically converted into a bladder acellular matrix graft in the lab. After the surgery, the patient was able to perform urination after the catheter removal. Also, no self-catheterization was necessary. The voiding intervals of the patient were increased, and the nocturia was reduced.

### Complications

The postoperative complications were reported in some patients in the included studies. The most reported complications included urinary infection, bladder stone formation, vesicoureteral reflux, bladder perforation, and bowel obstruction. These complications resolved spontaneously or with conservative management. No persistent complication was reported in the studies.

## Discussion

In the current systematic review, ten studies were retrieved, accounting for 63 patients. Five scaffold types were used in the included studies, including SIS (*n* = 4), biodegradable scaffolds seeded with autologous bladder muscle and urothelial cells (*n* = 3), dura mater (*n* = 1), bovine pericardium (*n* = 1), and human cadaveric bladder acellular matrix graft (*n* = 1). The scaffolds were used for patients with different disorders, including neurogenic bladder, spinal cord injury, myelomeningocele, exstrophy–epispadias complex, spina bifida, enterovesical fistula, and overactive contracted bladder. Overall, partially satisfying results for bladder reconstruction have been reached to date. However, SIS seems to have better results in relieving the patient’s symptoms and satisfaction. No serious or persistent postoperative complication was reported in the included studies. However, the human cadaveric bladder acellular matrix graft, bovine pericardium, and dura mater performed better regarding postoperative complications with no reported complications.

### Small intestinal submucosa

SIS, which is taken from the small intestine of pigs, is a xenogenic membrane, mostly acellular, water-proof, collagen-rich, and with no immunogenic effect [36, 41]. Using SIS, Caione et al. conducted bladder reconstructive surgery on five pediatric patients with small bladder capacity after exstrophy repair [36]. Despite the feasibility of bladder regeneration and its partial initial positive impact on bladder capacity, long-term significant clinical benefit in bladder capacity and compliance was not achieved [36]. There were several explanations for the relatively poor long-term outcomes, such as (a) inadequate increase in bladder capacity resulting in insufficient urinary continence and (b) imperfect morphometry of the regenerative wall histology (lower muscular fibers and higher collagen component compared with the native bladder) [36]. On the other hand, in a study by Zhang et al., cystoplasty using SIS resulted in improved bladder compliance and decreased intravesical pressure [35]. Furthermore, no complications regarding bowel function, renal function, or metabolism were observed. One of the patients completed the 36-month follow-up, showing the adequate capacity of the implanted bladder as well as preservation of renal function in the long term. However, since the sample size was small and not all of the subjects underwent long-term follow-up, the results are not considered translatable to the population of all patients with bladder disorders. In another study by Zhang et al., with a larger sample size (15 patients) and a longer follow-up period (4.5 to 8.3 y), an overall success rate of 60% was reported [34]. Major complications were bladder perforation in one patient, vesicoureteral reflux in five patients, and bladder stone formation in one patient [34].

### Autologous cell-seeded biodegradable scaffold

After obtaining a biopsy from each patient, muscle cells and urothelial cells are grown ex vivo and then seeded onto a biodegradable bladder-shaped scaffold to form a foundation for bladder tissue regeneration [31, 33]. This scaffold is called an autologous cell-seeded biodegradable scaffold [31]. One of the studied approaches for engineering bladder tissue is to utilize a composite scaffold consisting of polyglycolic acid for structural support and collagen for promoting cell growth and survival [31]. Moreover, in order to provide vascularization, due to its rich blood supply, omentum could be utilized as a wrap over the bladder [31, 42]. Therefore, the use of these composite scaffolds offers suitable biomechanical and structural characteristics required to maintain tissue integrity over an extended period. This approach was used in the trial by Atala et al. on seven patients with poorly compliant and high-pressure bladders, due to myelomeningocele. Using three implant types, including C, C + O, and PC + O, bladder compliance was increased significantly. However, in a similar study by Joseph et al. [33], conducting augmentation cystoplasty using autologous cell-seeded biodegradable scaffold in 10 patients with spina bifida, no statistically significant improvement was observed in bladder capacity [33]. Furthermore, only insignificant improvement in bladder compliance was reached in four and five patients at 12 months and 36 months postoperative period, respectively [33]. In another study by Shenot et al. [32], the implantation of neo-bladder augment (an autologous cell-seeded biodegradable scaffold) resulted in a partial success (two responders, two partial responders, and two non-responders, in baseline and two-year follow-up) among six patients with neurogenic bladder. Altogether, small sample sizes, disparities in the underlying diseases of patients, and differences in methodologies and follow-up times could explain the discrepancies in the results. Hence, future studies should be conducted using larger sample sizes, considering the limitations of the previous studies.

### Dura mater, bovine pericardium, and human cadaveric bladder acellular matrix graft

Another type of scaffold used in the studies is dura mater, serving as a matrix for bladder regeneration [38]. After a period of 10–12 weeks, it is absorbed, and the internal surface is completely epithelialized [43]. Using dehydrated human skull dura mater, Arikan et al. [38] conducted cystoplasty using dura mater on ten patients with neurogenic bladder dysfunction, which resulted in a relatively good success rate. Bovine pericardium [40] and Human cadaveric bladder acellular matrix graft [37] were also investigated in two case reports, resulting in satisfactory results. However, since the data in the two studies were merely for one patient in each, future studies with larger sample sizes are required in order to reach more reliable results.

### Limitations

The present study has several limitations. On the one hand, due to the heterogenicity of the studies and their data, a meta-analysis could not be performed. On the other hand, most of the included studies had small sample sizes; therefore, the findings cannot be generalized to the total population of patients with bladder dysfunction.

## Conclusions

Although the results of enterocystoplasties seem generally acceptable, they carry a number of possible complications, such as bladder replacements. In addition, using alternative treatment methods rather than enterocystoplasty showed relatively acceptable results in the assessed studies. Taken together, further studies and continued follow-up evaluations are required to concisely evaluate the response of patients to bladder replacements.

### Supplementary Information

**Additional file 1: Table S1.** : Search strategy used in databases.

## Data Availability

All relevant data are within the manuscript.
