# Supplementary material for: Current evidence regarding alternative techniques for enterocystoplasty using regenerative medicine methods: a systematic review
Source: Eur J Med Res. 2024 Mar 12;29:163. doi: 10.1186/s40001-024-01757-z (PMC10929228; doi:10.1186/s40001-024-01757-z)
Supplement: Supplementary file 1 — Additional file 1: Table S1. : Search strategy used in databases. [file 40001_2024_1757_MOESM1_ESM.docx]

**Table S1: Search strategy used in databases**

| PubMed | ("Tissue Scaffolds"[Mesh] OR "Tissue Scaffolds"[Title/Abstract] OR "Tissue Scaffold"[Title/Abstract] OR "Tissue Therapy, Historical"[Mesh] OR "Regenerative Medicine"[Mesh] OR "regenerative medicine"[Title/Abstract] OR "biocompatible materials"[Title/Abstract] OR "biomimetic materials"[Title/Abstract] OR "Tissue Engineering"[Mesh] OR "tissue engineering"[Title/Abstract] OR "Cell Engineering"[Mesh] OR "cell engineering"[Title/Abstract] OR "Bioengineering"[Mesh] OR "bioengineering"[Title/Abstract] OR "Biocompatible Materials"[Mesh] OR "biomaterial"[Title/Abstract] OR "biomaterials"[Title/Abstract] OR "bioartificial material"[Title/Abstract] OR "bioartificial materials"[Title/Abstract] OR "biocompatible material"[Title/Abstract] OR "biocompatible materials"[Title/Abstract] OR "hemocompatible material"[Title/Abstract] OR "hemocompatible materials"[Title/Abstract] OR "biomimicry"[Title/Abstract] OR "Biomimetic Materials"[Mesh] OR "Biomimetic Materials"[Title/Abstract] OR "Biomimetic Material"[Title/Abstract] OR "biomimicry materials"[Title/Abstract] OR "bioinspired material"[Title/Abstract] OR "bioinspired materials"[Title/Abstract] OR "bio-inspired material"[Title/Abstract] OR "bio-inspired materials"[Title/Abstract] OR "cellular engineering"[Title/Abstract] OR "biological engineering"[Title/Abstract] OR "Cell and Tissue-Based Therapy"[Mesh] OR "cell therapy"[Title/Abstract] OR "3d bioprinting"[Title/Abstract] OR "3d printing"[Title/Abstract] OR "Artificial Organs"[Mesh] OR "artificial organ"[Title/Abstract] OR "artificial organs"[Title/Abstract] OR "bioartificial organ"[Title/Abstract] OR "bioartificial organs"[Title/Abstract] OR "bioengineered material"[Title/Abstract] OR "bioengineered materials"[Title/Abstract] OR "Bioprinting"[Mesh] OR "bioprinting"[Title/Abstract] OR "tissue therapy"[Title/Abstract]) AND ("bladder reconstruction"[Title/Abstract] OR "neobladder"[Title/Abstract] OR "neobladder reconstruction"[Title/Abstract] OR "artificial bladder"[Title/Abstract] OR "neobladder"[Title/Abstract] OR "bladder augmentation"[Title/Abstract] OR "augmented bladder "[Title/Abstract] OR "bladder enlargement "[Title/Abstract] OR "cystoplasty"[Title/Abstract] OR "enterocystoplasty"[Title/Abstract] OR "vesicoplasty"[Title/Abstract] OR "neobladder" [Title/Abstract] OR "neobladders" [Title/Abstract] OR " bladder augmentation"[Title/Abstract] OR "tissue engineered bladder"[Title/Abstract] OR "bladder repair"[Title/Abstract] OR "bioengineered bladder"[Title/Abstract] OR "bladder plasty"[Title/Abstract]) |
| --- | --- |
| Embase | ('Tissue Scaffolds'/exp OR 'Tissue Scaffolds':ti,ab OR 'Tissue Scaffold':ti,ab OR 'Tissue Therapy, Historical'/exp OR 'Regenerative Medicine'/exp OR 'regenerative medicine':ti,ab OR 'biocompatible materials':ti,ab OR 'biomimetic materials':ti,ab OR 'Tissue Engineering'/exp OR 'tissue engineering':ti,ab OR 'Cell Engineering'/exp OR 'cell engineering':ti,ab OR 'Bioengineering'/exp OR 'bioengineering':ti,ab OR 'Biocompatible Materials'/exp OR 'biomaterial':ti,ab OR 'biomaterials':ti,ab OR 'bioartificial material':ti,ab OR 'bioartificial materials':ti,ab OR 'biocompatible material':ti,ab OR 'biocompatible materials':ti,ab OR 'hemocompatible material':ti,ab OR 'hemocompatible materials':ti,ab OR 'biomimicry':ti,ab OR 'Biomimetic Materials'/exp OR 'Biomimetic Materials':ti,ab OR 'Biomimetic Material':ti,ab OR 'biomimicry materials':ti,ab OR 'bioinspired material':ti,ab OR 'bioinspired materials':ti,ab OR 'bio-inspired material':ti,ab OR 'bio-inspired materials':ti,ab OR 'cellular engineering':ti,ab OR 'biological engineering':ti,ab OR 'Cell and Tissue-Based Therapy'/exp OR 'cell therapy':ti,ab OR '3d bioprinting':ti,ab OR '3d printing':ti,ab OR 'Artificial Organs'/exp OR 'artificial organ':ti,ab OR 'artificial organs':ti,ab OR 'bioartificial organ':ti,ab OR 'bioartificial organs':ti,ab OR 'bioengineered material':ti,ab OR 'bioengineered materials':ti,ab OR 'Bioprinting'/exp OR 'bioprinting':ti,ab OR 'tissue therapy':ti,ab) AND ('bladder reconstruction':ti,ab OR 'neobladder':ti,ab OR 'neobladder reconstruction':ti,ab OR 'artificial bladder':ti,ab OR 'neobladder':ti,ab OR 'bladder augmentation':ti,ab OR 'augmented bladder':ti,ab OR 'bladder enlargement':ti,ab OR 'cystoplasty':ti,ab OR 'enterocystoplasty':ti,ab OR 'vesicoplasty':ti,ab OR 'neobladder':ti,ab OR 'neobladders':ti,ab OR 'bladder augmentation':ti,ab OR 'tissue engineered bladder':ti,ab OR 'bladder repair':ti,ab OR 'bioengineered bladder':ti,ab OR 'bladder plasty':ti,ab) |
| Cochrane Library | "Tissue Scaffolds" OR "Tissue Scaffold" OR "Tissue Therapy, Historical" OR "Regenerative Medicine" OR "biocompatible materials" OR "biomimetic materials" OR "Tissue Engineering" OR "Cell Engineering" OR "Bioengineering" OR "Biocompatible Materials" OR "biomaterial" OR "biomaterials" OR "bioartificial material" OR "bioartificial materials" OR "biocompatible material" OR "biocompatible materials" OR "hemocompatible material" OR "hemocompatible materials" OR "biomimicry" OR "Biomimetic Materials" OR "Biomimetic Material" OR "biomimicry materials" OR "bioinspired material" OR "bioinspired materials" OR "bio-inspired material" OR "bio-inspired materials" OR "cellular engineering" OR "biological engineering" OR "Cell and Tissue-Based Therapy" OR "cell therapy" OR "3d bioprinting" OR "3d printing" OR "Artificial Organs" OR "artificial organ" OR "artificial organs" OR "bioartificial organ" OR "bioartificial organs" OR "bioengineered material" OR "bioengineered materials" OR "Bioprinting" OR "bioprinting" OR "tissue therapy"  AND "bladder reconstruction" OR "neobladder" OR "neobladder reconstruction" OR "artificial bladder" OR "bladder augmentation" OR "augmented bladder" OR "bladder enlargement" OR "cystoplasty" OR "enterocystoplasty" OR "vesicoplasty" OR "neobladder" OR "neobladders" OR "bladder augmentation" OR "tissue engineered bladder" OR "bladder repair" OR "bioengineered bladder" OR "bladder plasty" |
